# Active Adoption of Void Formation in Metal-Oxide for All Transparent Super-Performing Photodetectors

**DOI:** 10.1038/srep25461

**Published:** 2016-05-06

**Authors:** Malkeshkumar Patel, Hong-Sik Kim, Hyeong-Ho Park, Joondong Kim

**Affiliations:** 1 Department of Electrical Engineering, Incheon National University, 119 Academy Rd. Yeonsu, Incheon, 406772, Republic of Korea; 2 Applied Device and Material Lab., Device Technology Division, Korea Advanced Nanofab Center (KANC), Suwon 443270, Korea

## Abstract

Could ‘defect-considered’ void formation in metal-oxide be actively used? Is it possible to realize stable void formation in a metal-oxide layer, beyond unexpected observations, for functional utilization? Herein we demonstrate the effective tailoring of void formation of NiO for ultra-sensitive UV photodetection. NiO was formed onto pre-sputtered ZnO for a large size and spontaneously formed abrupt p-NiO/n-ZnO heterojunction device. To form voids at an interface, rapid thermal process was performed, resulting in highly visible light transparency (85–95%). This heterojunction provides extremely low saturation current (<0.1 nA) with an extraordinary rectifying ratio value of over 3000 and works well without any additional metal electrodes. Under UV illumination, we can observe the fast photoresponse time (10 ms) along with the highest possible responsivity (1.8 A W^−1^) and excellent detectivity (2 × 10^13^ Jones) due to the existence of an intrinsic-void layer at the interface. We consider this as the first report on metal-oxide-based void formation (Kirkendall effect) for effective photoelectric device applications. We propose that the active adoption of ‘defect-considered’ Kirkendall-voids will open up a new era for metal-oxide based photoelectric devices.

Ultraviolet (UV) radiation (290–400 nm) has been looked upon as an important segment only encompasses less than 10% of the solar radiation. UV radiation has a profound impact on the survival and development of humankind. For example, moderate skin exposure to natural or artificial UV light is advantageous for health, for instance, facilitating the synthesis of Vitamin D, killing germs, treating or preventing rickets, etc. By contrast, excess exposure to UV light induces immune suppression. Exposure of the human skin to UV from sunlight results in deleterious effects on the skin such as sunburn, immune suppression, photo-aging, and skin cancer. Also, UV radiation influences the output of crops and the lifespan of buildings^1^,^2^,^3^. Most UV radiation can be absorbed by stratospheric ozone, and it has been monitored since the early 1980s. Recently, anthropogenic decline in stratospheric ozone concentration simultaneously intensifying UV radiation, has become alarming, and the ‘ozone hole’ over Antarctica has become larger^4^,^5^,^6^,^7^. Therefore, the study of UV detection has drawn immense interest among researchers^3^,^8^,^9^,^10^,^11^,^12^,^13^,^14^,^15^,^16^.

State of the art reviews of UV photodetectors are available in the literature, including reviews of new concept ultraviolet photodetectors^3^,^8^,^9^,^10^. With continuous advances in materials and device architecture, UV photodetectors have come to be effectively used in communications, flame detection, air purification, ozone sensing, and leak detection, among other areas, in the past few decades. However, with the growing need for practical applications, researchers are attempting to exceed the performance of conventional UV photodetectors and, possibly, integrate such devices with existing platforms. For the moment, low cost, large area, transparent, smart, flexible, low-powered, reliable, and air stable UV photodetectors are urgently needed in fields of health, infrastructure, and environment monitoring, especially for unattended stations^17^,^18^,^19^,^20^,^21^,^22^,^23^,^24^,^25^. ZnO based UV photodetectors based on low dimensional nanostructures have been rigorously studied in the past few decades^3^,^8^,^9^,^10^. Including new materials that exist at nanoscale, excellent figure of merit parameters (gain^13^,^20^,^26^, detectivity^13^, responsivity^13^,^27^, linear dynamic range^28^, transient photoresponse^29^, and thermal stability^30^) have been obtained. Largely, low dimensional UV photodetectors are composed of various materials, such as ZnS, Nb_2_O_3_, GaO_3_, In_2_O_3_, SnO_2_, GaN, ZnO, V_2_O_5_, In_2_Se_3_, InSe, CdS, CdSe, ZnSe, Sb_2_Se_3_, ZrS_2_, Ag_2_S, and Zn_x_Cd_1−x_Se, which involve expensive and toxic starting materials or complex fabrication processes for large scale applications^31^,^32^. Importantly, the usually slow photoresponse of these UV photodetectors is a monumental obstruction in practice.

Heterojunction architecture offers the inherent advantage of wide spectral range absorption; high speed carrier transport can be facilitated by interface engineering and low power operation compared to that used in conventional UV nanoscale photoconductors. To gain the advantage of the heterostructure of the widely studied material ZnO^3^,^8^,^9^,^10^,^33^,^34^,^35^, wide band gap (3.7 eV) and intrinsically p-type NiO could be a worthwhile material for the development of a low cost, non-toxic, and high performing all-transparent metal oxide UV detector. Therefore, NiO/ZnO heterojunctions have been fabricated using various techniques and have been intensively studied for UV detection performance enhancement over the past couple of years^36^,^37^,^38^,^39^. Slow photoresponse is retarding the advancement of UV photodetectors.

Here, we report a novel highly transparent heterostructure for high speed UV photodetection. The sputtering method was applied to deposit n-type ZnO and p-type NiO layers in sequence (Supplementary Fig. 1)^40^,^41^,^42^. The structural, physical, optical, and electrical properties of nanocrystalline NiO and ZnO have been systematically investigated. Developed devices were scrutinized for NiO thickness optimization. The optimally thick nanoscaled NiO with ZnO exhibited high photodetection with a high rectification ratio and visible blind functional operation without any additional signal processing amplification. This p-NiO/n-ZnO photodetector can work well without an external metal electrode and showed excellent UV photoresponse among the NiO-based UV photodetectors^39^. Air operation photodetection functionality analysis of various parameters (NiO thickness, applied bias, and UV intensity) has confirmed the high stability and reproducibility.

The working mechanism of the presented NiO/ZnO heterostructure was found to be an important one for metal oxide photoelectric devices. Our analysis discovered that voids embedded in the nanocrystalline NiO/ZnO interface play a pivotal function in high speed UV photodetection. The formation of a continuum of voids at the NiO/ZnO interface was systematically explained by considering the Kirkendall effect, in which pores form because of a difference in diffusion rates between two components in a diffusion couple. The supersaturation of the faster diffusion element may lead to a condensation of extra vacancies in the form of so-called “Kirkendall voids” close to the interface^43^,^44^. A profound UV photodetection mechanism that can be obtained if Kirkendall voids act as an ideal insulating barrier backing the effective tunneling of photogenerated free carriers is proposed here. We demonstrate that active adoption of Kirkendall void defects will greatly enhance the performance of metal-oxide photoelectric devices and thus open up an era of defect-assisted functional applications.

## Results

**Figure 1:**
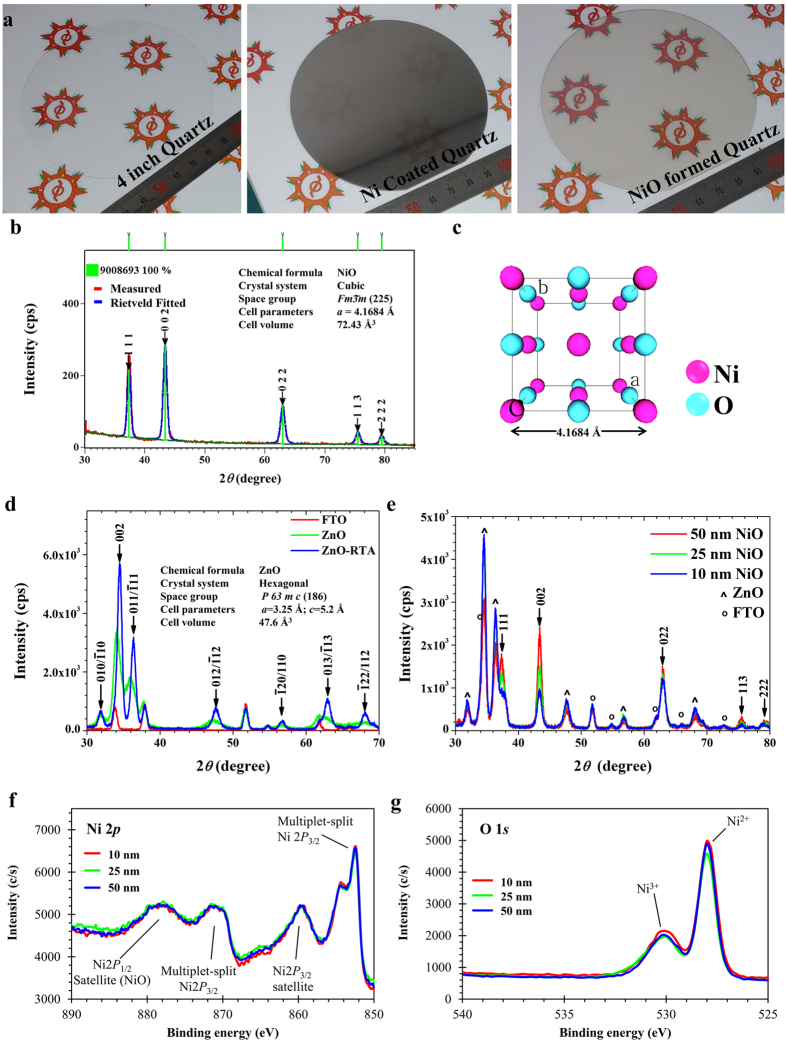
(**a**) Transparent NiO film in large scale (Ø4 inch), (**b**) Glancing-angle X-ray diffraction pattern of nanocrystalline NiO film along with quantitative fitting profile, (**c**) Perspective view of the cubic unit cell of the NiO material obtained from the Rietveld analysis, (**d**) XRD patterns of the ZnO on the FTO substrate, and (**e**) XRD patterns of the nanocrystalline NiO with various thicknesses on the ZnO/FTO substrate. XPS results of nanocrystalline NiO films, (**f**) Ni 2*p*_3/2_, and (**g**) O 1*s* core level peaks.

Large scale (4 inch)-available NiO film can be achieved as shown in Fig. 1a. X-ray diffraction (XRD) pattern of NiO film from thermal oxidation of the sputtered Ni film, along with a quantitative structural fitting profile is shown in Fig. 1b. All of the experimental XRD peaks match the standard COD-AMCSD 9008693 for NiO with cubic structure (space group: *Fm-3m*(225)), with the various plane assignments shown in Fig. 1b. Quantitative structural analysis was performed by Rietveld refinement of the XRD patterns using HighScorePlus software. The unit cell of the observed cubic nanocrystalline NiO has an average crystallite size of approximately 25 nm (calculated from peak broadening of the (002) peak using the Scherrer relation). The obtained crystal structure of the nanoscaled NiO had an ideal lattice parameter of *a* = 4.1684 Å and is shown in Fig. 1c. This confirms the successful conversion of Ni into nanocrystalline NiO using simple ambient rapid thermal processing (RTP).

We also found that RTP is significantly effective to crystallize the bottom-ZnO layer, which corresponds to the more intense and narrow peak appearance shown in Fig. 1d. The XRD spectra of nanoscale NiO films with various thicknesses from 10 nm to 50 nm are shown in Fig. 1e, revealing the distinct structural properties of FTO and ZnO films during RTP of 20 minute duration. Furthermore, the 50 nm thick NiO remained nanocrystalline (All the Bragg planes for NiO, ZnO, and FTO were observed and denoted in the respective XRD spectra in Fig. 1b,d,e).

The obtained X-ray photoelectron spectroscopy results corresponding to Ni 2*p* and O 1*s* for various thickness of NiO are shown in Fig. 1f,g, respectively (Also refer Supplementary Figs 2–4 for various NiO thicknesses). XPS analysis of the nanocrystalline NiO with various thicknesses revealed non-significant differences at the surfaces in the peak shape and intensity, except for the O 1*s* peak at 529.8 eV, which corresponds to the Ni^3+^ state and is attributed to the higher supply of oxygen compared to the available amount of Ni during RTP^45^. In the present case, the processing parameters may favor the formation of an extremely thin Ni_2_O_3_ layer on the surface of nanocrystalline NiO^45^,^46^. This layer could be detrimental for charge transport in device applications.

**Figure 2:**
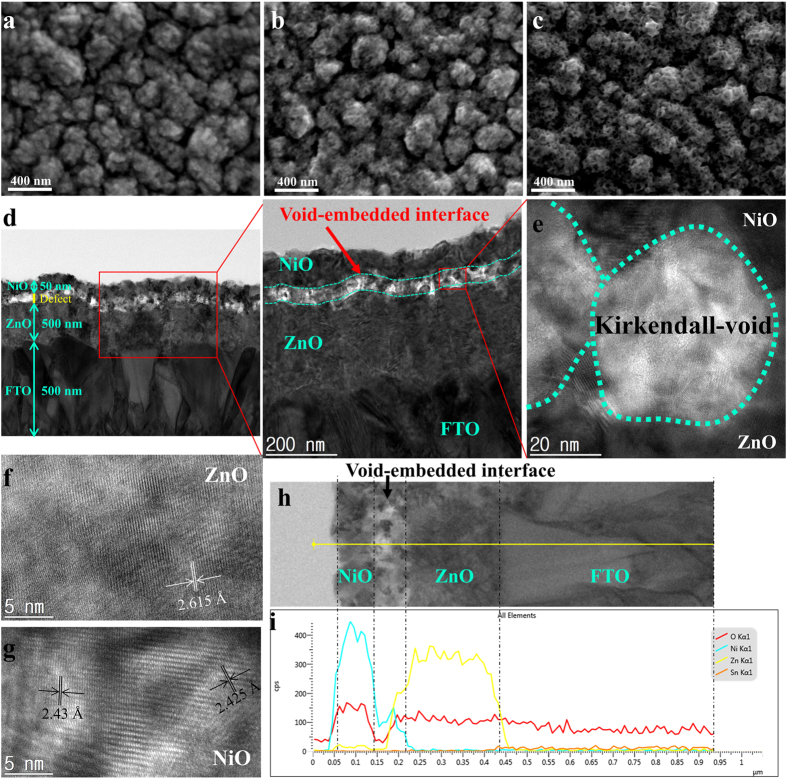
FESEM images of topography of nanocrystalline NiO with various thicknesses (**a**) 17 nm, (**b**) 34 nm, and (**c**) 50 nm. (**d**) Cross-sectional TEM image NiO/ZnO heterojunction indicating the defective interface; the continuum empty space was found between the ZnO and NiO layers. (**e**) The enlarged section reveals spherical void formation. (**f**,**g**) HRTEM images of highly crystalline ZnO and nanocrystalline NiO, respectively. (**h**,**i**) Cross-sectional HRTEM image and elemental line mapping using energy dispersive spectroscopy, respectively.

We prepared several samples with varying of NiO thickness (*t*_*NiO*_) from 8–50 nm; these samples are denoted as D1-D6 and their properties are summarized in Table 1. The surface morphology, as seen in the field emission scanning electron microscopy (FESEM) images taken at 5 k× (Supplementary Fig. 5) and 50 k× (Fig. 2a–c) of magnification reveal a compact and uniformly interconnected NiO nanocrystalline structure on the FTO layer. We found that the formed spherical nanocrystalline NiO becomes distinct, compact, and porous as the thickness increases from 8 nm to 50 nm. The applied process facilitates tailoring of the nanocrystalline NiO. The acquired FESEM images of nanoscaled NiO exhibited substantial flat field intensity distribution over the surface morphology; this flatness was observed for all samples without any additional charging effect (Please refer to Supplementary Fig. 5). It was revealed that surface of NiO is a conducting one, with a homogeneous free carrier distribution. Mott-Schottky analysis revealed that nanocrystalline NiO has a free accepter carrier concentration of the order of 10^18 ^cm^−3^. It is noteworthy to mention that no pin holes were observed on the surface for all samples (before and after RTP), demonstrating the promising nature of this approach to functional and high quality metal oxide layers for large scale applications (Fig. 1, Supplementary Figs 1c and 5).

It is known that the thermal oxidation process for nanoscaled Ni particles can be implemented by diffusion phenomena; consequently, core shell hollow NiO particles are likely to form^47^,^48^. Herein, we studied the interface properties of heterostructured nano NiO at the ZnO surface using high resolution transmission electron microscopy (HRTEM). HRTEM images of heterostructured NiO/ZnO (Fig. 2d,e) reveal an interface with empty voids. Continuum void formation was found at the nanoscaled NiO and ZnO interface. According to the Kirkendall effect, voids are likely to form at the inside of the dynamic hetero interface and then grow until conversion is complete^43^,^49^,^50^,^51^. The number of voids and the growth mechanisms are dependent on the relative rates of self-diffusion in the core material (Ni) versus the cation diffusion through the shell (NiO)^47^. HRTEM analysis revealed the formation of spherical (*ϕ* = 20–30 nm) voids at the interface of ZnO and NiO due to RTP assisted oxidation. These voids are attributed to the higher self-diffusion rate of Ni than that of Ni^2+^ through NiO. The estimated *d*-spacing values of 2.43 Å and 2.615 Å correspond to the ZnO (002) plane (Fig. 2f) and the NiO (111) plane (Fig. 2g), respectively.

Elemental line profiles across the cross-section (Fig. 2h,i) of the novel NiO/vacuum (Kirkendall void)/ZnO interface confirm the absolute oxidation of the Ni species, following the Kirkendall effect. The elemental profiles of O, Ni, Zn, and Sn reveal the formation of 25 nm diameter void formation, which is also clearly evident in the visual HRTEM (Fig. 2e). Moreover, these spherical Kirkendall voids were formed at a continuum 20–30 nm interfacial layer at the NiO/ZnO interface (Please refer to Supplementary Fig. 6), hence, we can consider the NiO/ZnO interface as a spherical space (with Kirkendall voids) embedded in an interfacial continuum.

Our HRTEM analysis revealed three important facts about the observed novel NiO/vacuum/ZnO heterostructure. First, nanocrystalline NiO does not contain hollow spherical Kirkendall voids (Supplementary Fig. 7), which voids are likely to form in cases of using an oxidation process for Ni nanoparticles and diffusion of Ni^2+^ from the core (Ni) to the shell (NiO) until the conversion ends^43^,^47^,^48^. Second, the occupancy of spherical voids is in the NiO layer (Supplementary Fig. 8). Third, the existence of spherical Kirkendall voids forms the continuum interfacial layer at the NiO/ZnO interface (Please refer to Fig. 2d,e and Supplementary Fig. 6).

**Figure 3:**
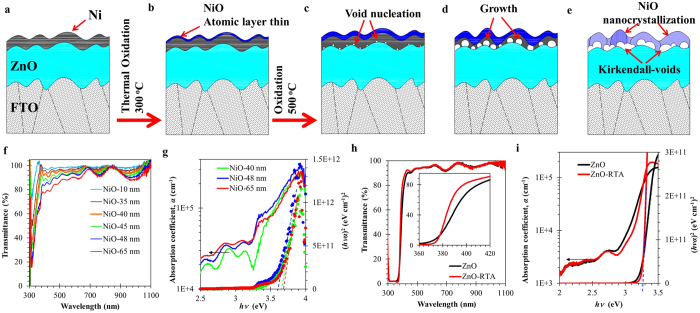
Illustration of the observed formation of spherical voids at the interface of NiO and ZnO, attributed to the self-diffusion of Ni from bottom of Ni layer through formed NiO layer following Kirkendall phenomena. (**a**) Prior to thermal oxidation process, 20 nm of Ni film was coated, (**b**) formation of uniform 3–5 nm thick NiO layer triggers the self-diffusion of Ni from bottom, (**c**) diffused Ni forms uniform voids at the surface of ZnO, (**d**) voids grow until conversion is complete, (**e**) NiO layer followed by the nanocrystallizations. Optical properties of the developed nanocrystalline NiO and highly crystalline ZnO. (**f**) Transmittance spectra of nanoscaled NiO has at various thicknesses from 10 nm to 65 nm, and (**g**) absorption coefficient and Tauc plot of NiO films. (**h**) Transmittance spectra of ZnO (inset shows the effected region where annealing process improves the transition nature of transmittance property), and (**i**) absorption coefficient and Tauc plot of ZnO films before and after RTA.

According to these observations, we can propose the growth sequence of the novel Kirkendall-void embedded NiO/ZnO heterostructure, as illustrated in the Fig. 3a–e. The Kirkendall void formation is composed of four steps. 1) Formation of a few thin atomic layers of NiO on the Ni surface due to oxidation process at 300 °C. 2) Activation of Ni oxidation at an elevated temperature of 500 °C to generate multiple void nucleation at ZnO/NiO interface due to Ni cation diffusion. 3) Acceleration of growth of Kirkendall voids for complete Ni conversion to form the continuum interfacial layer. 4) Crystallization of NiO layer^47^.

The optical properties of NiO layers were investigated for transmittance and absorption tendencies, with results shown in Fig. 3f,g, respectively. Nanoscaled NiO possesses excellent transmittance greater than 90% for visible and near infrared regions; however, the high absorption coefficient in the UV-A region makes this material excellent for optoelectrical devices with an additional transparent function. That nanoscale NiO offers the highest possible transparency to date^52^, is due to the spherical nanocrystalline NiO^40^,^53^,^54^. The absorption coefficient, *α,* is estimated using the following relation:


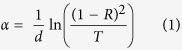


where *d*, *R,* and *T* are the film thickness, reflectance, and transmittance of the film, respectively (*d* values of NiO and ZnO films were estimated using surface profiler analysis and are shown in Supplementary Fig. 9). The *α* values are plotted as a function of the photon energy (*E* = *hν*) in Fig. 3g, where *h* and *ν* are Planck’s constant and the photon frequency, respectively. Tauc analysis has revealed an energy band gap of 3.6–3.7 eV for 40–65 nm thick NiO film (secondary axis of Fig. 3g).

Our observations show promising improvement in the optical properties of ZnO influenced by the RTP oxidation of Ni. The values of *T* and *α* of the ZnO film before and after RTP processing are shown in Fig. 3h,i. These figures show improvements in the values of *T* (please refer to the inset in Fig. 3h) and of the absorption transition (Fig. 3i); however, the energy bandgap of ZnO remained at 3.25 eV for both samples (from the Tauc plot, secondary axis of Fig. 3i). It is noteworthy to mention that the improved values of transition rate of absorption at the bandgap and of transmittance in the visible region may be solely attributed to the enhanced crystallite packing density of ZnO (Fig. 1d)^55^,^56^. The adopted ambient RTP process for the formation of NiO on ZnO has the potential to produce high quality transparent ultraviolet functional solid state devices. Furthermore, the integrated optical properties of NiO and ZnO films may lead to the production of visible transparent high-performance heterojunction devices for functional detection of UVA.

**Figure 4:**
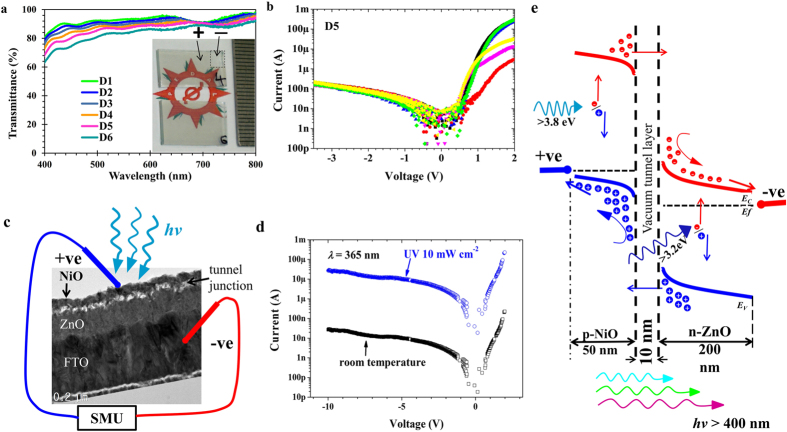
(**a**) Transmittance feature of the developed all metal oxide heterojunction device. (**b**) Room temperature dark current-voltage characteristics of the D5 batch of devices, (**c**) the device under test setup with cross sectional presentation of developed nanocrystalline NiO/ZnO UV photodetector with 15 nm thick intrinsic tunnel layer. (**d**) Current-voltage characteristics with visible blind operation at room temperature and under 10 mW cm^−2^ of intense UV light. Device possesses excellent signal to noise ratio value of 1000. (**e**) The energy band diagram represents the p-i-n tunnel junction NiO/Vacuum/ZnO photodetector in the reverse bias operation with visible transparent feature.

Figure 4a shows the transmittance spectra of the novel void embedded NiO/ZnO heterojunction, for which the thicknesses of NiO were varied (device image is given in the inset). Transmittances of less than 4% in the UV-A region and greater than 80% in the visible region were obtained for the D4 (33 nm) batch NiO heterojunction devices. The current-voltage (*I*-*V*) characteristics of all the devices for batches D1-D6 (Table 1) were studied at room temperature by placing (manually) a tungsten probe on the nanoscaled NiO surface. The obtained results are shown in Fig. 4b and Supplementary Fig. 10. All samples demonstrate diode properties with high rectification ratio and extremely low saturation current. Device possesses low saturation current of less than 1 nA. The estimated diode properties for all devices are summarized in the Table 1; the diode ideality factor (*IF*) was calculated according to the relation


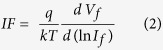


where *q*/*kT*, and *dV*_*f*_/*d*(ln*I*_*f*_) are the thermal voltage and the slope of *ln*(*I*_*f*_) vs. *V* plotted at very low forward voltage. The devices in batch D5 showed the best diode properties (Table 1), with an *IF* value of 1.3 and a rectification ratio (*RR*) value of 3417. In order to understand the extremely low saturation current (*I*_*O*_) and the *RR*, we have tried to correlate these values with the void embedded NiO/ZnO structure. These values could be the result of voids assisting the tunneling in the forward bias condition and, on the other hand, blocking the carrier transport in the reverse bias condition. Hence, from this point we will consider the embedded voids as an intrinsic layer and propose a resultant solid state structure with the configuration of the Kirkendall void-embedded p(NiO)-n(ZnO) heterostructure.

The novel Kirkendall void-embedded heterojunctions have shown significant photoresponse when exposed to UV light with a wavelength of 365 nm and various levels of intensity (Supplementary Fig. 11). All devices showed photoresponse except for D1. Devices of batches D2-D6 were found to be highly sensitive to very low levels of UV light intensity (20 μW cm^−2^).

Figure 4c provides an operational illustration of the void embedded NiO/ZnO photodetector under a measurement setup connected with a source measurement unit (SMU), in which a nanocrystalline NiO surface was probed with tungsten and connected to a positive terminal and, on the other hand, FTO was connected to a negative terminal. The obtained *I*-*V* characteristics results are shown in Fig. 4d and indicate an excellent photoresponse with a signal to noise ratio of more than 1000. The detailed energy band diagram of the Kirkendall void embedded NiO/ZnO UV detector in the applied reverse bias condition is carefully developed and shown in Fig. 4e. It can be seen in the band diagram that when the device is exposed to UV light, the combination of NiO and ZnO can absorb the entire UV-A region and consequently generate electron hole pairs. However, due to the high barrier that results from Kirkendall void formation at the interface of the NiO and ZnO surfaces, these photogenerated carriers are not able to pass the Kirkendall voids without bias. The introduced vacuum layer could be the result of cross-through tunneling if these photogenerated carriers can obtain adequate kinetic energy through drift assisted transport at the interface. On the other hand, the majority of the photogenerated carriers of ZnO and NiO can effectively be collected at the collection terminal. Moreover, the developed novel Kirkendall-void embedded NiO/ZnO has less recombination probability for photogenerated free carriers at the junction if the device is biased in the reverse condition (photodetection mode operation). Because we know that heterojunctions are the majority carrier device, the large fraction of photogenerated current under reverse bias condition is attributed to drift-assisted carrier transport (holes in NiO and electrons in ZnO). The Kirkendall-void continuum has an important role in high speed UV detection because it enables minority photogenerated carriers to cross the interface very quickly via tunneling and join the majority carriers. In general, for a semiconductor surface in which the lattice is disrupted, recombination is a very crucial concern. Consequently, increased interface surface due to Kirkendall voids at the NiO/ZnO interface facilitate high recombination rate when UV light is turned off. Furthermore, we have scanned the reverse bias condition up to −10 V under dark and UV light (365 nm, 10 mW cm^−2^) conditions using SMU.

**Figure 5:**
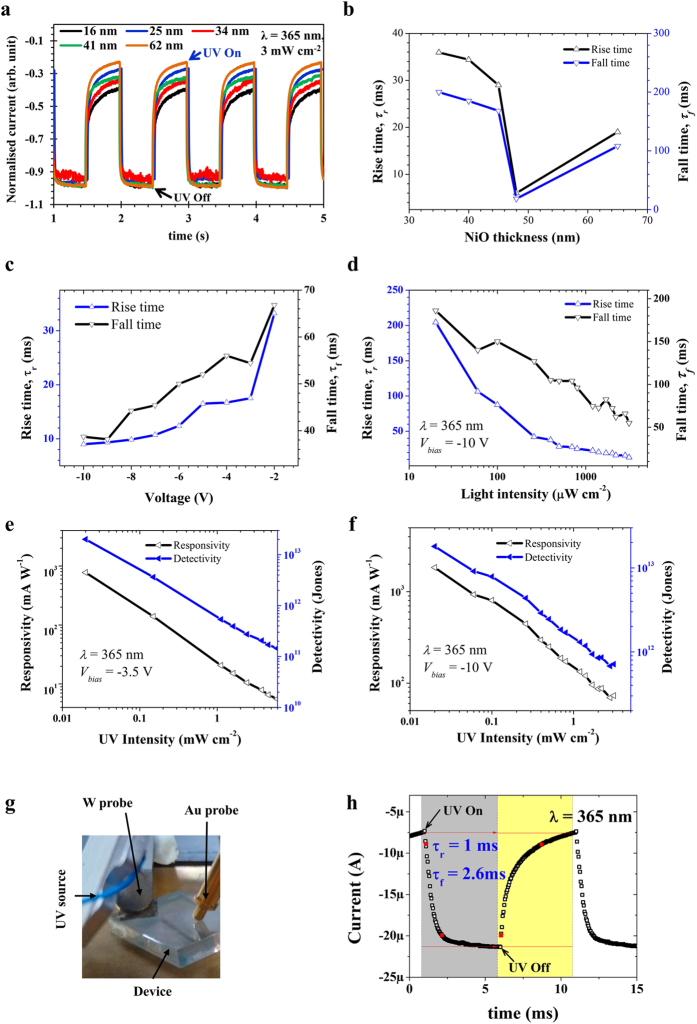
(**a**) The effect of NiO layer thickness on the photoresponse (The normalized values of the obtained photocurrent were compared for all devices), and (**b**) rise and fall time as a function of NiO thickness. Rise and fall time as a function of (**c**) bias voltage, and (**d**) UV light intensity. Plots of responsivity and detectivity for light intensity modulation for different bias conditions, (**e**) −3.5 V, and (**f**) −10 V. Transient photoresponse characteristics with Au probe, (**g**) setup, and (**h**) rise and fall time.

To evaluate the photoresponse speed of the transparent Kirkendall-void embedded NiO/ZnO UV photodetector, the rising and falling edges were carefully analyzed. Figure 5a shows the normalized photoresponse for various thicknesses of the NiO photodetector. Rise (*τ*_*r*_) and fall (*τ*_*f*_) times are defined as the time required to change from 10% to 90% and from 90% to 10% of the peak output value of the photodetector, respectively. The estimated values of *τ*_*r*_ and *τ*_*f*_ are summarized in Fig. 5b and in Table 1; it was found that these values are a function of the nanoscaled NiO thickness. Careful analyses of the photoresponse curves for all devices are shown in Supplementary Fig. 12. The sample denoted D5 (44 nm-thick NiO) was found to be the optimum one to obtain the highest *τ*_*r*_ and *τ*_*f*_ values of 6 ms and 18 ms, respectively, for UV light detection (365 nm wavelength with 3mW cm^−2^). This is the fastest reported UV photodetection among all NiO/ZnO UV detectors to date^39^. The slow response time and poor photoresponse of UV detection remain challenges for the implementation of wide bandgap UV detection devices^19^,^26^,^29^,^31^. It is worthwhile to note that this novel Kirkendall-void embedded p-NiO/n-ZnO heterojunction may allow the realization of the promise of transparent wide bandgap UV devices with high quality junction formation for high speed and stable photoresponse.

Furthermore, the applied bias (−2 V to −10 V) and UV light intensity (20 μW cm^−2^ to 3.1 mW cm^−2^) dependent photoresponse of the devices of the D5 (44 nm thick NiO) batch were carefully examined. Supplementary Fig. 13 shows the photoresponse values acquired at various levels of reverse bias from −2 to −10 V for a UV light wavelength of 365 nm; these values reveal excellent photoresponses with stable performance. We found that the values of *τ*_*r*_ and *τ*_*f*_ are highly bias voltage dependent for void embedded NiO/ZnO UV detectors. The values of *τ*_*r*_ and *τ*_*f*_ as a function of applied reverse bias are shown in Fig. 5c (Supplementary Fig. 14).

Supplementary Fig. 15 shows the photoresponse acquired for UV light of 365 nm at various levels of intensity from 20 μW cm^−2^ to 3.1 mW cm^−2^, revealing an excellent photoresponse (fixed −10 V of applied bias). The values of *τ*_*r*_ and *τ*_*f*_ are found to be highly UV light intensity dependent for void the embedded NiO/ZnO UV detector. The values of *τ*_*r*_ and *τ*_*f*_ as a function of UV light intensity are shown in Fig. 5d (Supplementary Fig. 16, in which the magnified regions of interest are shown). According to our observations (Fig. 5c,d), as bias and light intensity increase, *τ*_*r*_ decreases logarithmically and tends toward a saturation value as low as 8 ms for an applied bias of −10 V and light intensity of 3.1 mW cm^−2^. However, for the cases of applied bias and light intensity, *τ*_*f*_ follows a linear trend. It is noteworthy to mention that the Kirkendall-void embedded NiO/ZnO UV photodetector is highly stable under atmospheric operating conditions.

To investigate the figure of merit, responsivity (R^*^) and detectivity (D^*^) are the key parameters for performance evaluation of a photodetector. R^*^ specifies the efficiency of the detector as it responds to optical signals; this value is given by the relation: 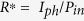, where *I*_*ph*_ and *P*_*in*_ are the photocurrent and light intensity, respectively. Meanwhile, the detectivity denotes the ability of the photodetector to detect weak optical signals. D^*^ can be calculated from the relation: 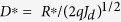, where *J*_*d*_ is the background current density^36^. According to this D^*^ relationship, a low background current results in a high detectivity value. The R^*^ and D^*^ values of the Kirkendall-void embedded transparent NiO/ZnO UV photodetector were studied by recording the *I*-*V* characteristics under UV light (λ = 365 nm) with intensity modulation from 20 μW cm^−2^ to 3.1 mW cm^−2^ for two different biases of −3.5 V and −10 V; the results are shown in Fig. 5e,f. It is worthwhile to note that the Kirkendall-void embedded NiO/ZnO is highly sensitive to very low level UV light (20 μW cm^−2^) conditions. Moreover, the quality of the interface formation of the Kirkendall-void embedded NiO/ZnO facilitates lossless transport of the photogenerated carriers under reverse bias conditions. Obviously, both R^*^ and D^*^ are more sensitive to low UV intensity. The estimated values of R^*^ and D^*^ for the nanocrystalline NiO UV detector were as high as 1.8 × 10^3^ mA W^−1^ and 2 × 10^13^ Jones for 365 nm-UV excitation at 20 μW cm^-2^ intensity. This is the highest responsivity so far achieved for UV photodetection by all transparent metal oxide photodetectors produced to date^10^,^19^,^24^,^39^. The Kirkendall-void embedded NiO/ZnO UV photodetector has demonstrated a considerable linear dynamic range (*LDR)* value of ~54 dB, which may allow the realization of ultraviolet high contrast imaging (please refer to Supplementary Fig. 17).

Finally, high speed photoresponse of the Kirkendall-void embedded NiO/ZnO photodetector was obtained when the tungsten tip was replaced with an Au tip on the NiO surface. Figure 5g,h provide photographs of the setup with the Au probe and magnified areas of interest to estimate the photoresponse time (*τ*_*r*_ and *τ*_*f*_), respectively. The estimated *τ*_*r*_ and *τ*_*f*_ values of 1 ms and 2.6 ms for the proposed Kirkendall-void embedded NiO/ZnO photodetector allow the conception of real time quick ultraviolet photodetection for next generation transparent photoelectric devices.

## Discussion

In summary, we have reported a novel all-metal oxide-based transparent high speed UV photodetector. The promise of large energy bandgap metal oxide semiconductor materials has been realized in a visible light transparent UV sensor. A potentially large scale sputtering method was employed to deposit p-type NiO and n-type ZnO in sequence; this deposition spontaneously established an abrupt junction, resulting in a high quality heterostructured solid state photoelectric device. The oxidation process, which was optimized by varying the Ni thickness to allow for a fixed thermal processing parameter, has revealed the consistent formation of high purity nanocrystalline NiO. Furthermore, the structural and optical properties of ZnO were found to have improved during the NiO formation. Among the metal-oxide based photodetectors, the optimal 45 nm thick p-NiO/n-ZnO UV photodetector showed the fastest photoresponse time (6 ms), without any opaque metal electrodes. Moreover, the repeatability and stability were also proven to be excellent.

Kirkendall-void embedded nanocrystalline metal oxide interfacial continuum formation was observed due to the Kirkendall-effect driven oxidation process. This phenomenon was observed for the first time in heterojunction metal oxide thin films and this is the first device level investigation that has been comprehensively presented. State of the art UV detection properties were systematically studied and it was found that Kirkendall-void embedded NiO/ZnO enables the tunneling of photogenerated minority carriers and effectively collects the photogenerated majority carriers. Remarkably fast response times of 1 ms (rise time) and 2.6 ms (fall time) were achieved when the tungsten probe was replaced with a gold probe on the nanoscaled NiO surface. We for the first time have demonstrated that the formation of ‘Kirkendall voids’ can be used for practical and high functional photodetector applications. And, we expect that active adoption of ‘defect-considered’ Kirkendall voids will open up a new era for metal oxide based photoelectric devices.

## Methods

### Device Fabrication of UV photodetector

The UV photodetector has a structure of NiO/ZnO/FTO-coated glass. A commercial fluorine-doped tin oxide (FTO)-coated glass (735167, Sigma Aldrich) was used as a substrate, with a sheet resistance of 7 Ω/□. This FTO-coated glass was chemically cleaned by a sequence of isopropyl alcohol, acetone, and distilled water using ultrasonication. After this, a 250 nm thick ZnO film was deposited on an FTO-coated glass using an RF sputtering system. RF power (3.58 W/cm^2^) was applied to a 4-inch ZnO target (purity, 99.99%). To form the NiO film above the ZnO film, Ni (purity, 99.999%) was deposited by DC sputtering method with power density of 3.70 W/cm^2^. A rapid thermal process (RTP) was performed at 500 ± 5 °C in an air ambient by flowing oxygen (0.8 LPM, liter per minute) for 20 minutes to transform the Ni into NiO. Batches of NiO/ZnO device were produced. Crystalline Si wafers were used as substrate for RTP processing to obtain uniform thermal conduction for oxidation of sputtered Ni thin film. The thickness of NiO was varied from 8 nm to 60 nm for photodetection performance optimization. The device fabrication process flow and batch distribution, including the process parameters, are shown in Supplementary Fig. 1a,b, respectively. The batches produced before and after rapid thermal processing are shown in Supplementary Fig. 1c.

### Characterization of ZnO and NiO films

The crystal structure of the NiO film was characterized by X-ray diffraction (XRD, Rigaku, SmartLab) with Cu-*K*_*α*_ radiation (*λ*_*Kα*_ = 1.540598 Å) in Grazing mode with glancing angle of 1.2°. The planar morphologies were analyzed using a field emission scanning electron microscope (FESEM, JEOL, JSM_7800F) with 5 kV of field voltage, using an SE2 secondary detector.

### Characterization of NiO/ZnO/FTO layers

In order to observe the crystalline structures of NiO, ZnO, FTO, and the interfaces, a field-emission transmission electron microscope (FETEM, JEOL, JEM-2100F) was used. Cross-sectional TEM samples were prepared using a focused ion beam system (FIB, FEI, Quanta 3D FEG). The elemental compositions as line profile in the cross section of the various layers in the device were determined by an energy dispersive spectroscopy (EDS) attachment to the FETEM. The thickness and average surface roughness of the deposited films were characterized using a surface profiler (Vecco, Dektak XT-E). Optical characterization was carried out using a UV-visible spectrophotometer (Shimadzu, UV-1800) by recording the transmission and absorbance spectra of the thin films in the range 300–1100 nm.

### Measurements of the UV photodetector

Room temperature dark *I*-*V* measurements were performed using a source meter unit (Keithley, 2400). The photoresponse characteristics were studied using a Potentiostat/Galvanostat from WonA Tech (model, ZIVE SP1). The tungsten probe was employed for making contact with the NiO surface. Photoresponses of the UV photodetector were obtained using a quantum measurement system (K3100, McScience) with on and off pulses of light from a 365 nm monochromatic LED lamp. The LED source was calibrated with a power meter (KUSAM-MECO, KM-SPM-11). All measurements were performed without any intentional external amplification.

## Additional Information

**How to cite this article**: Patel, M. *et al.* Active Adoption of Void Formation in Metal-Oxide for All Transparent Super-Performing Photodetectors. *Sci. Rep.* **6**, 25461; doi: 10.1038/srep25461 (2016).

## Supplementary Material

Supporting Information

## Figures and Tables

**Table 1:** Summary of nanoscaled NiO/ZnO/FTO heterojunction device performance parameters.

Code	*t*_*NiO*_ (nm)	*dV*/*dln*(*I*)	*I*_*O*_ (nA)	*RR*	*IF*	*τ*_*r*_ (ms)	*τ*_*f*_ (ms)
D1	8	0.0859	1.5	209	3.32	–	–
D2	17	0.0893	7.0	139	3.45	36	200
D3	25	0.1124	3.2	123	4.35	34	185
D4	33	0.0386	5.9	44	1.49	29	200
D5	44	0.0339	5.2	3417	1.31	6	18
D6	50	0.0361	5.5	876	1.40	19	108

Here, *t*_*NiO*_, *I*_*O*_, *RR*, and *IF* are thickness of NiO layer, dark saturation current, rectification ratio, and diode ideality factor, respectively. *RR* was estimated at +2 V.
