# Supplementary material for: Active Adoption of Void Formation in Metal-Oxide for All Transparent Super-Performing Photodetectors
Source: Sci Rep. 2016 May 6;6:25461. doi: 10.1038/srep25461 (PMC4858702; doi:10.1038/srep25461)
Supplement: Supporting Information [file srep25461-s1.pdf]

# Supporting Information

Active Adoption of Void Formation in Metal-Oxide for All Transparent Super-Performing Photodetectors

Malkeshkumar Patel,[1] Hong-Sik Kim,[1] Hyeong-Ho Park,[2] and Joondong Kim[1]*

[1] Department of Electrical Engineering, Incheon National University, 119 Academy Rd. Yeonsu, Incheon, 406772, Republic of Korea
[2] Applied Device and Material Lab., Device Technology Division, Korea Advanced Nanofab Center (KANC), Suwon 443270, Korea

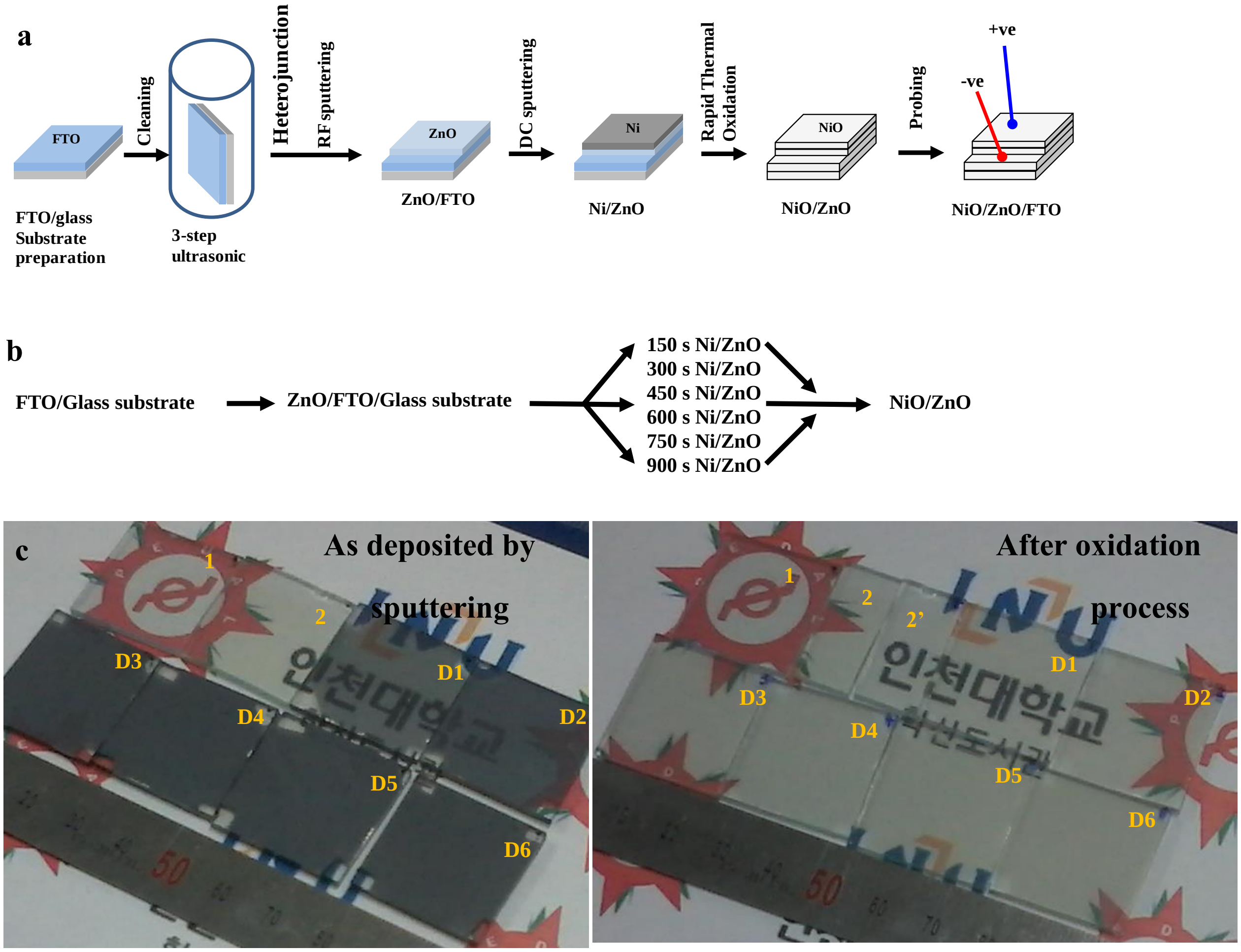


| | | | |
|---|---|---|---|
| 1 | FTO (500 nm)/Glass | | |
| 2 | ZnO (250 nm)/FTO/Glass | D3 | NiO(25 nm)/ZnO(250 nm)/FTO/Glass |
| 2' | ZnO (250 nm)/FTO/Glass – RTA processed | D4 | NiO(33.3 nm)/ZnO(250 nm)/FTO/Glass |
| D1 | NiO(8.3 nm)/ZnO (250 nm)/FTO/Glass | D5 | NiO(41.7 nm)/ZnO(250 nm)/FTO/Glass |
| D2 | NiO(16.7 nm)/ZnO (250 nm)/FTO/Glass | D6 | NiO(50 nm)/ZnO(250 nm)/FTO/Glass |

**Supplementary Figure 1.** (**a**) Device fabrication process flow.(**b**) Batch distribution, and (**c**) Photograph of produced batch of devices and sample coding.

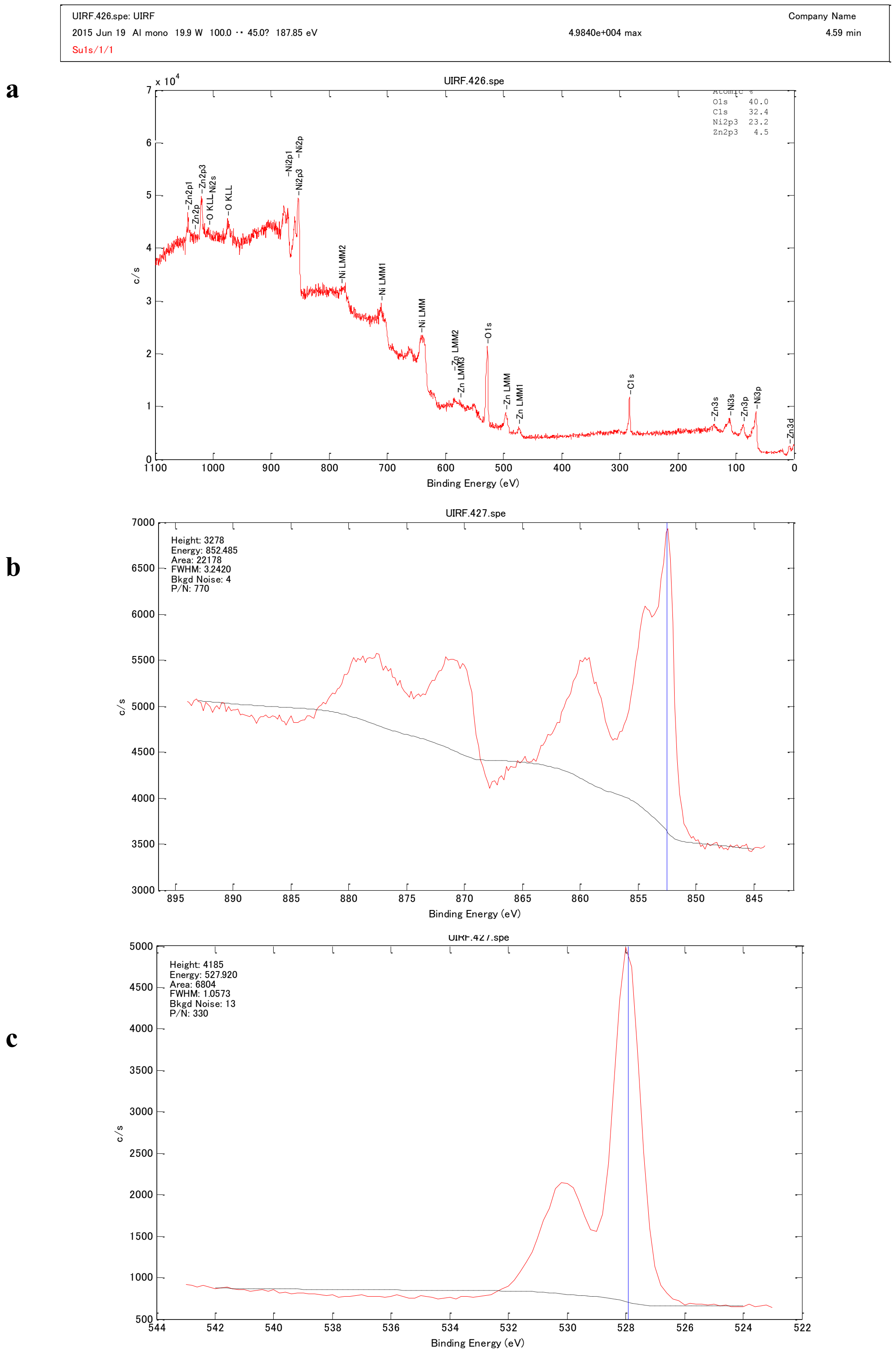


**Supplementary Figure 2.** XPS spectra of 17 nm (D2) thick nanocrystalline NiO. (**a**) Entire spectrum, (**b**) Ni 2p region, and (**c**) O 1s region.

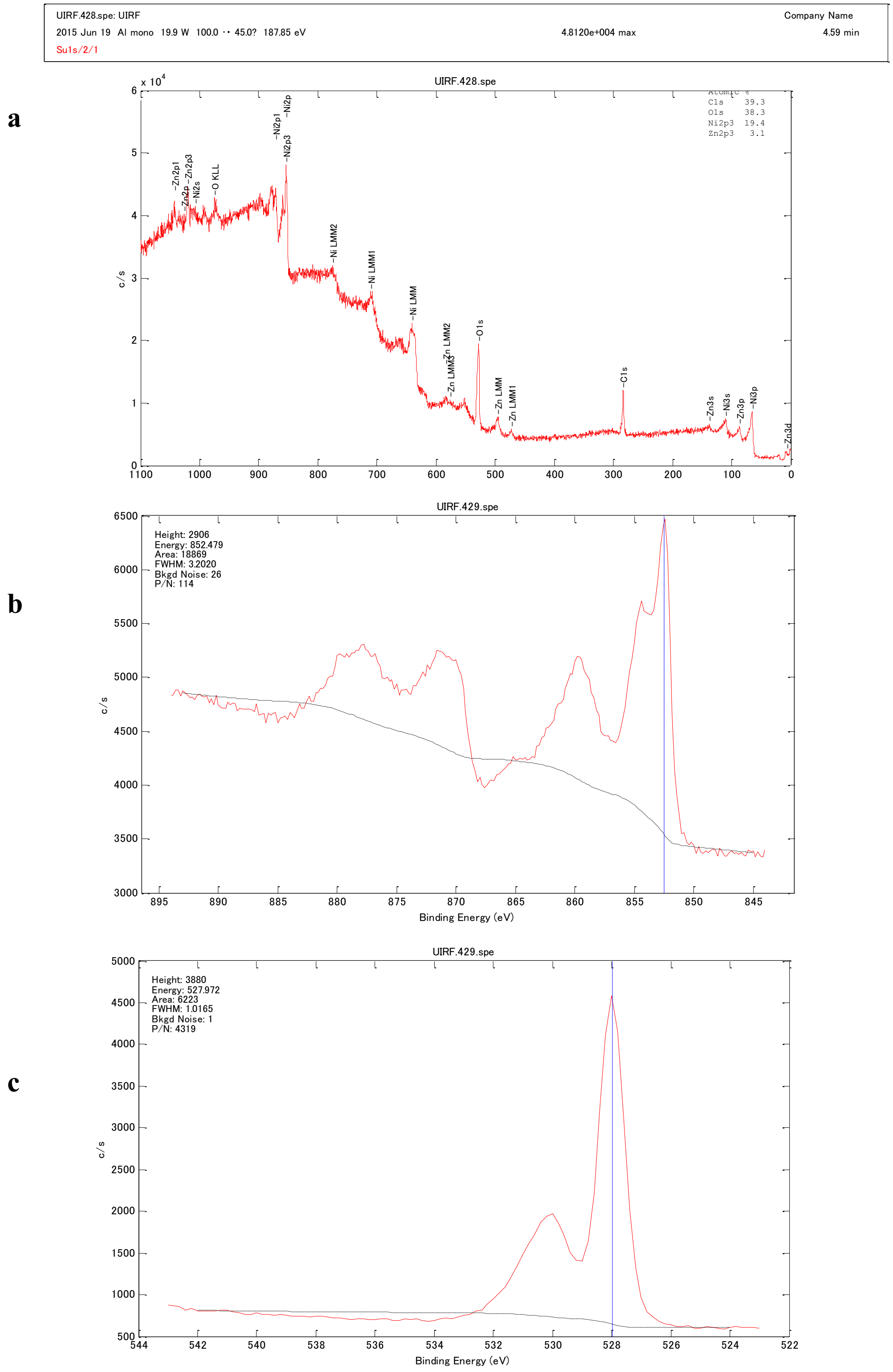


**Supplementary Figure 3.** XPS spectra of 33.3 nm (D4) thick nanocrystalline NiO. (**a**) Entire spectrum, (**b**) Ni 2p region, and (**c**) O 1s region.

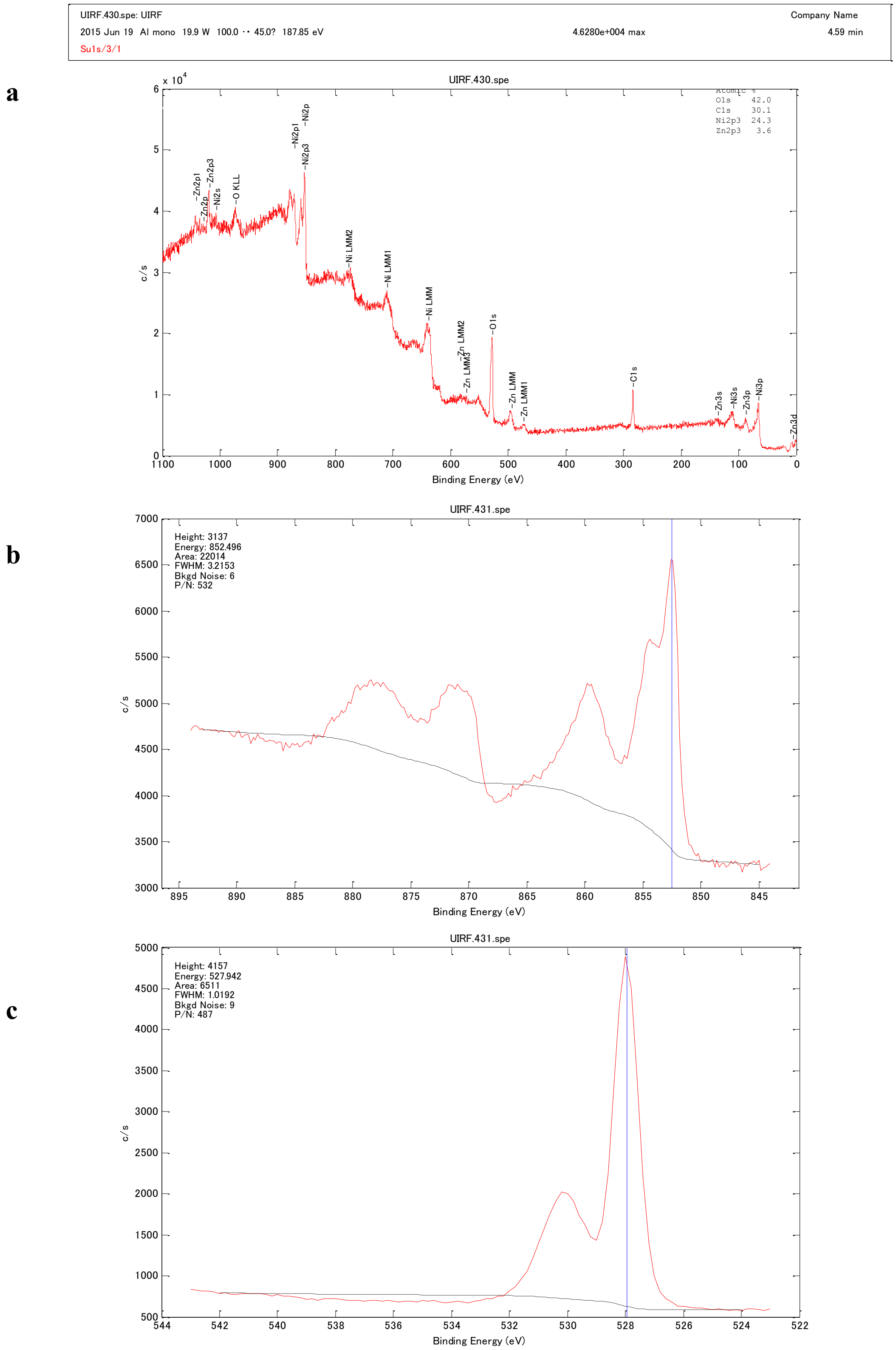


**Supplementary Figure 4.** XPS spectra of 50 nm (D6) thick nanocrystalline NiO. (**a**) Entire spectrum, (**b**) Ni 2p region, and (**c**) O 1s region.

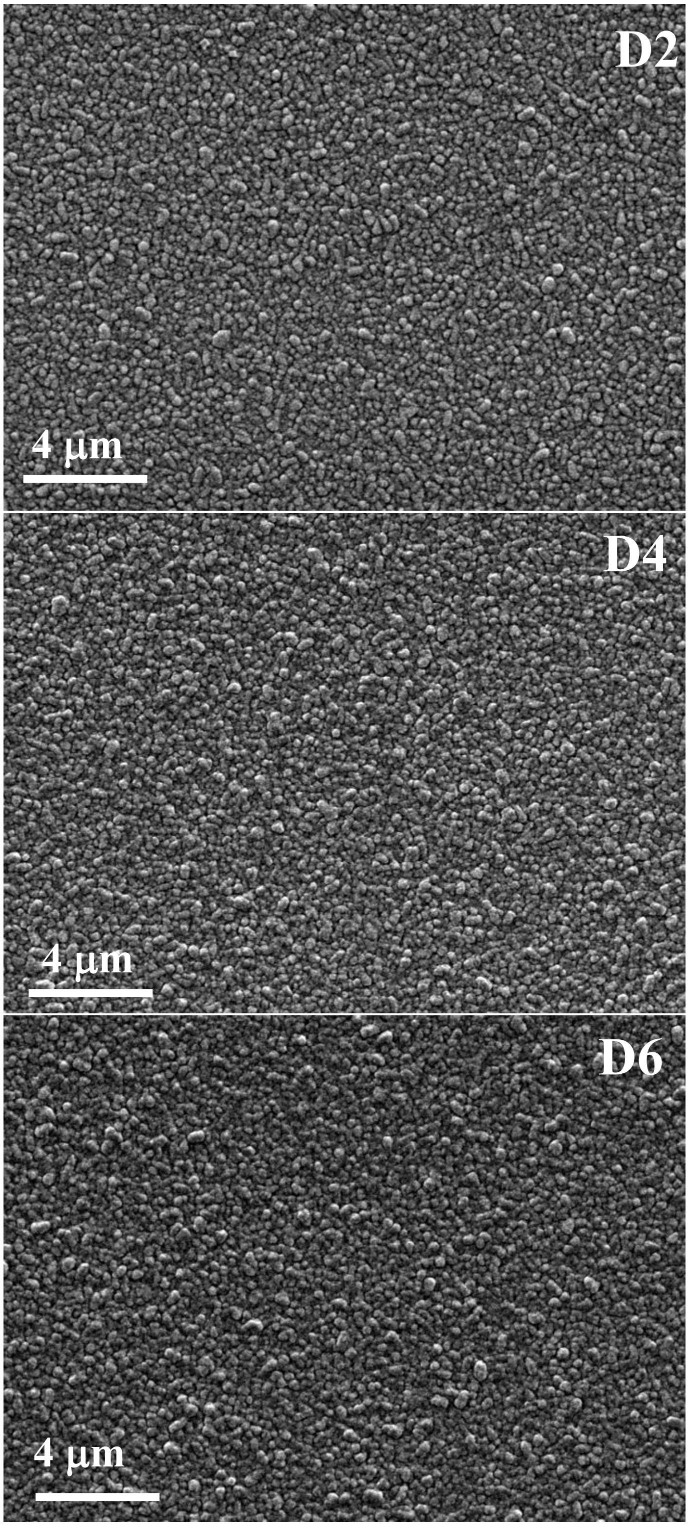


**Supplementary Figure 5.** FESEM images of topography of nanocrystalline NiO at lower magnification of 5 k×.

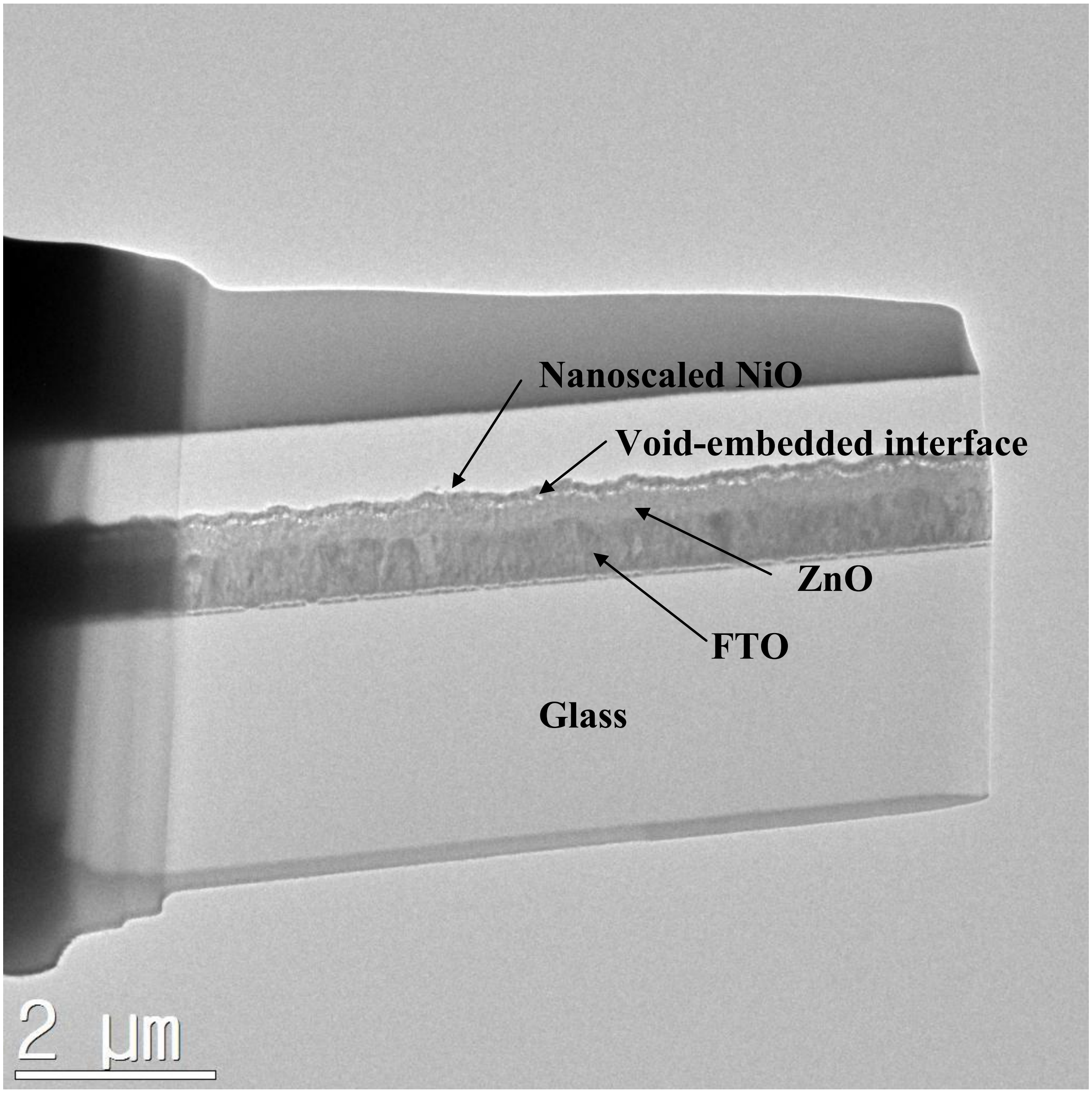


**Supplementary Figure 6.** Low resolution TEM image for sample prepared using focused ion beam confirms the formation of continuum interfacial layer at the interface of NiO/ZnO heterostructure.

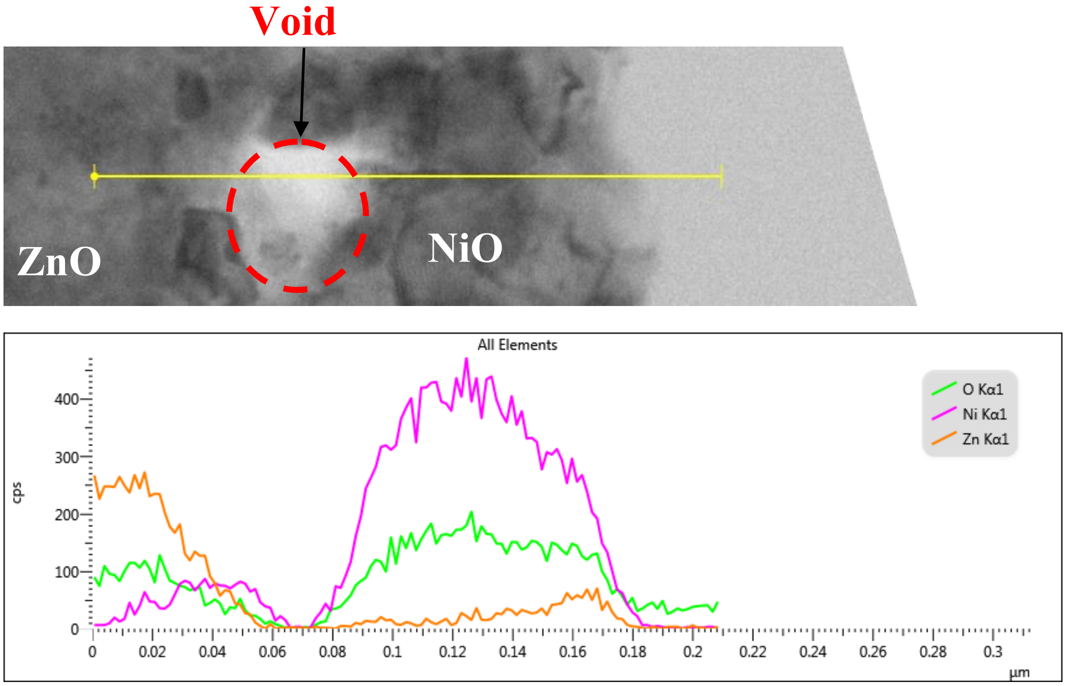


**Supplementary Figure 7**. Selected area TEM at the interface (ZnO/Void/NiO), where void are grown completely, and respective elemental line profile.

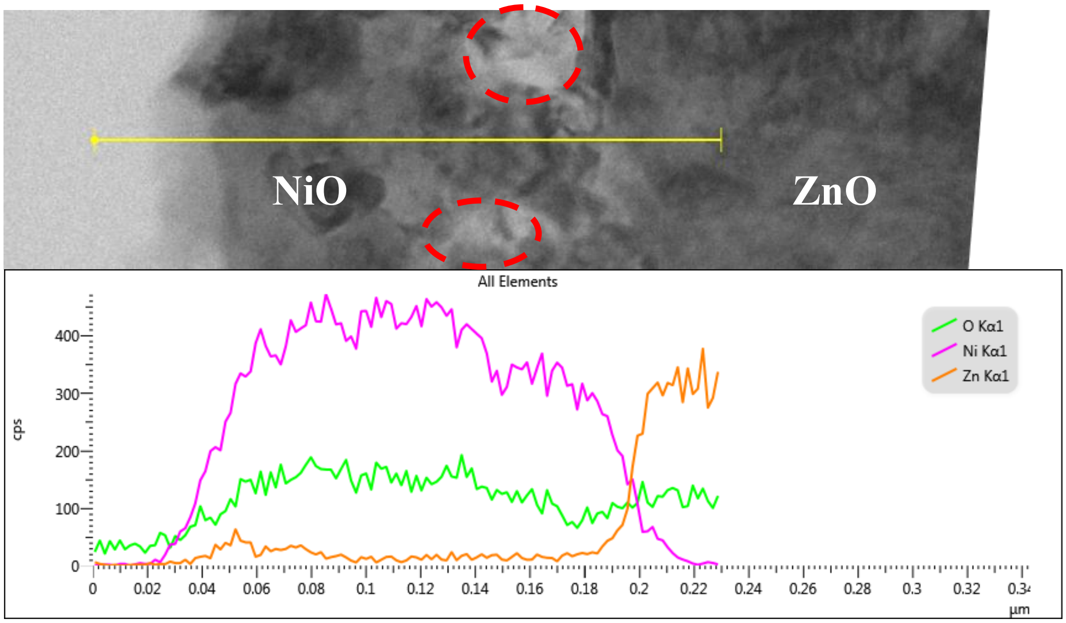


**Supplementary Figure 8.** Selected area TEM at the interface (NiO/ZnO), and respective elemental line profile.

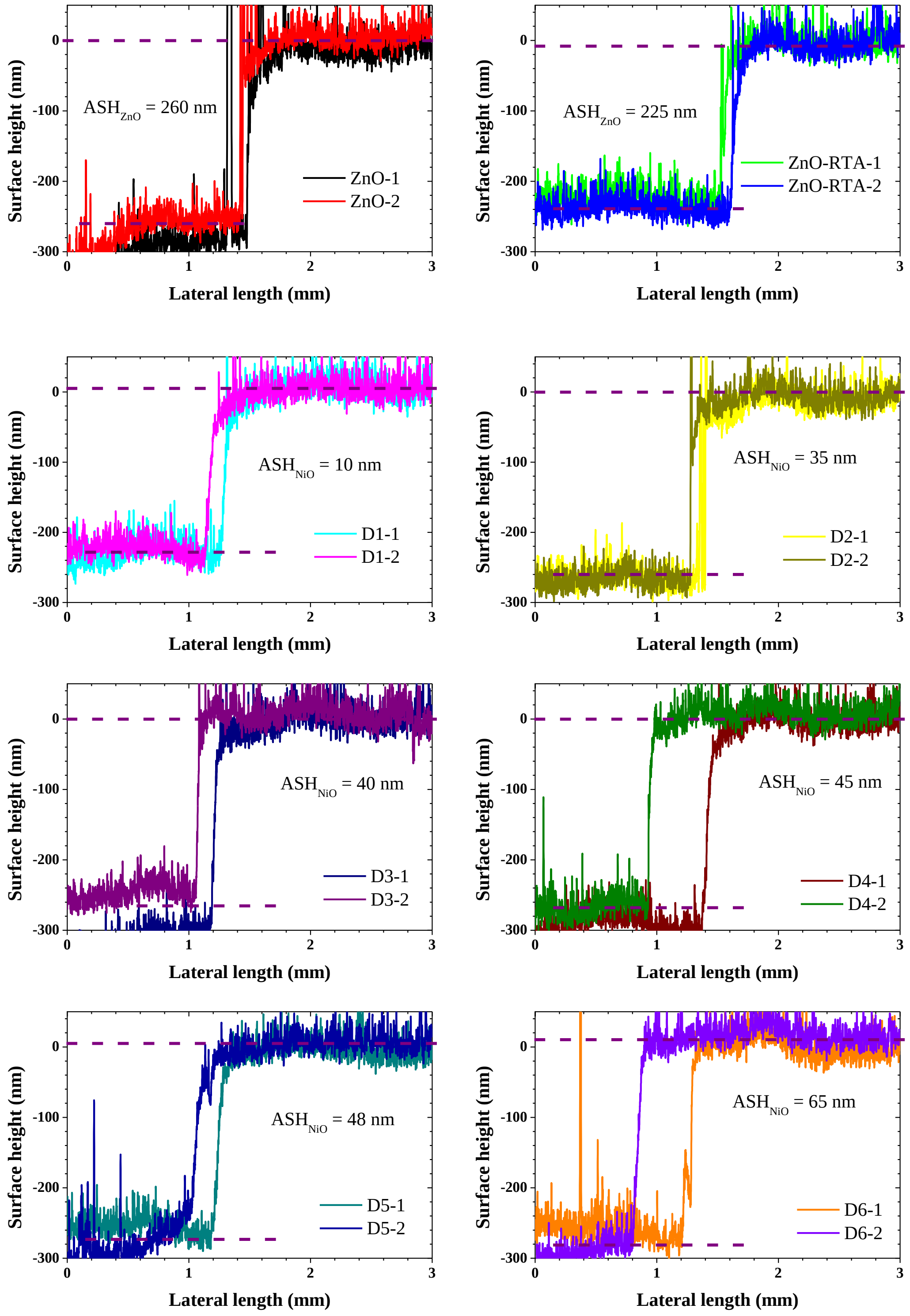


**Supplementary Figure 9.** Surface profiler analyses of device and films before and after oxidation process. The average surface height (ASH) is estimated and noted in the inset of each graph.

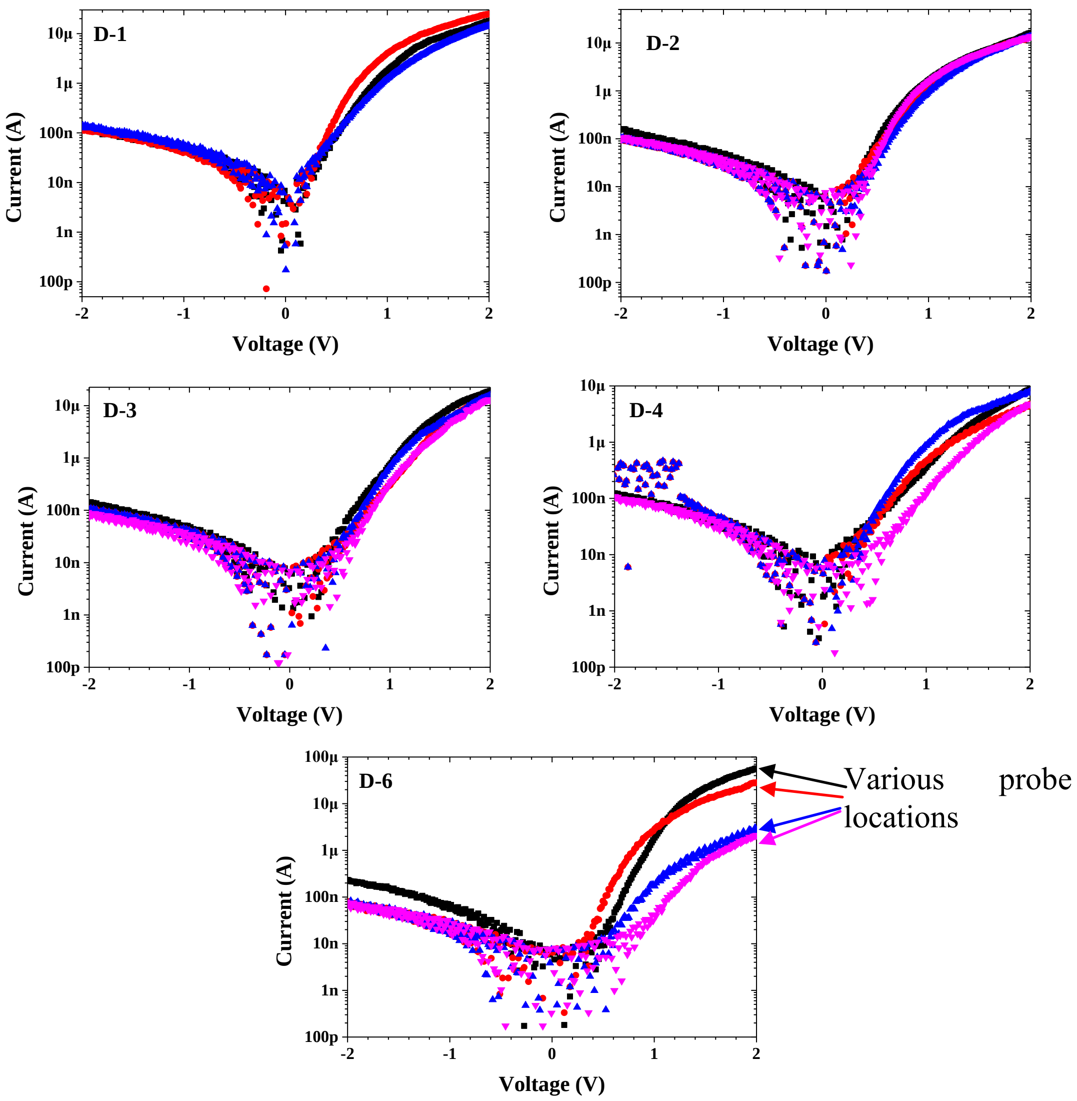


**Supplementary Figure 10.** Room temperature current-voltage characteristics of the developed nanoscaled NiO/ZnO transparent heterojunction devices (D1-D4, D6).

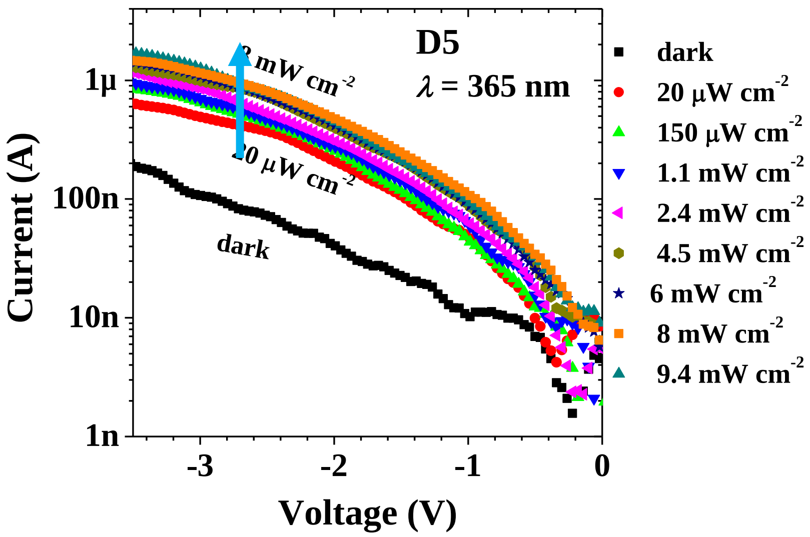


**Supplementary Figure 11.** Photoresponse characteristics of the heterojunction measured under UV light with various light intensities in the range of 20 μW $cm^{-2}$ to 9.4 mW $cm^{-2}$ in the photodiode region.

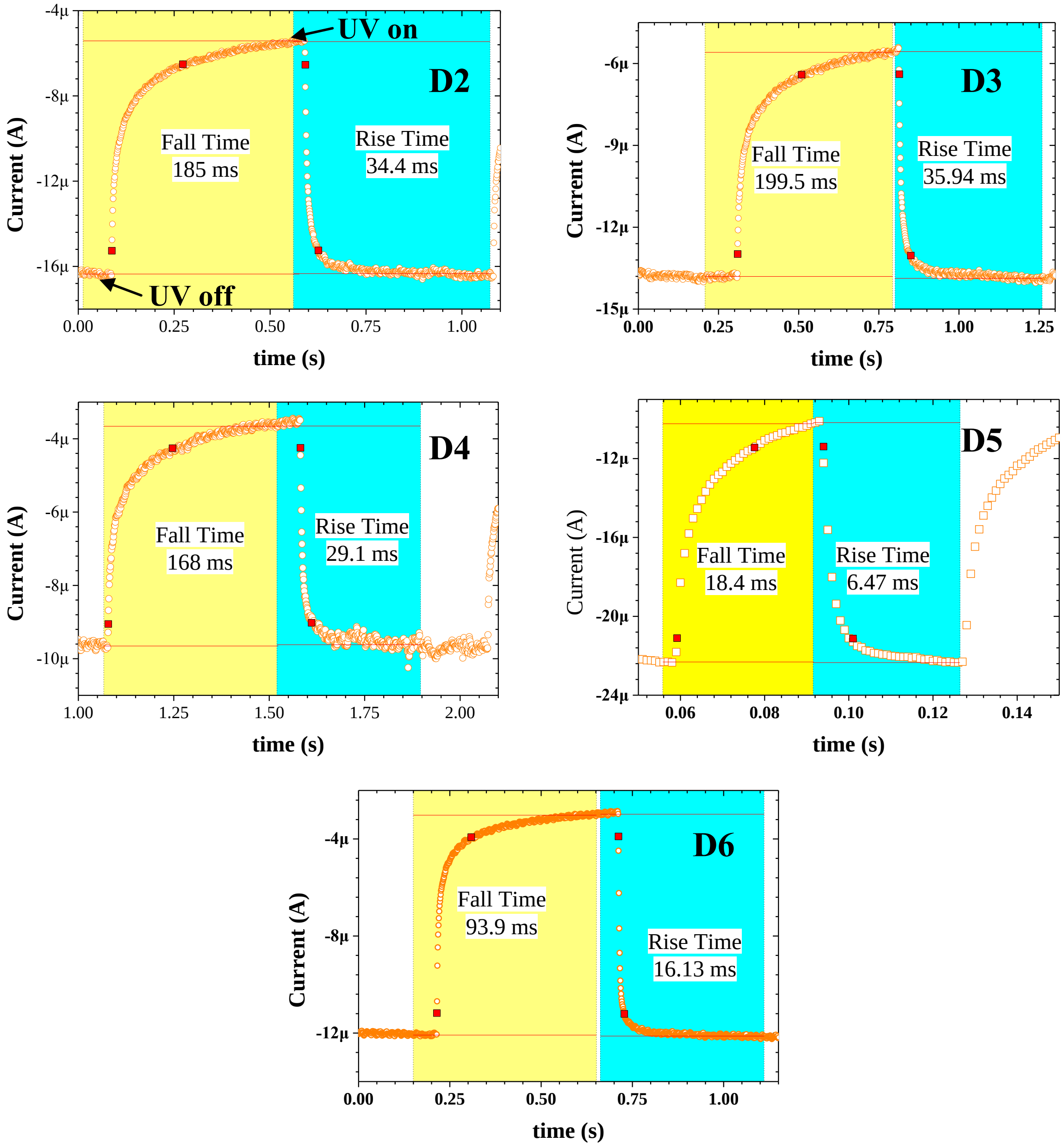


**Supplementary Figure 12.** The magnified rise and fall edges of the photoresponse of devices D1-D6 at bias of -10 V. The rise time, $\tau_r$, is the time interval for the response to rise from 10% to 90% of its peak value. The fall time, $\tau_f$, is the time interval for the response to decay from 90% to 10% of its peak value. UV source has a wavelength of λ = 365 nm and intensity of 3.1 mW cm$^{-2}$.

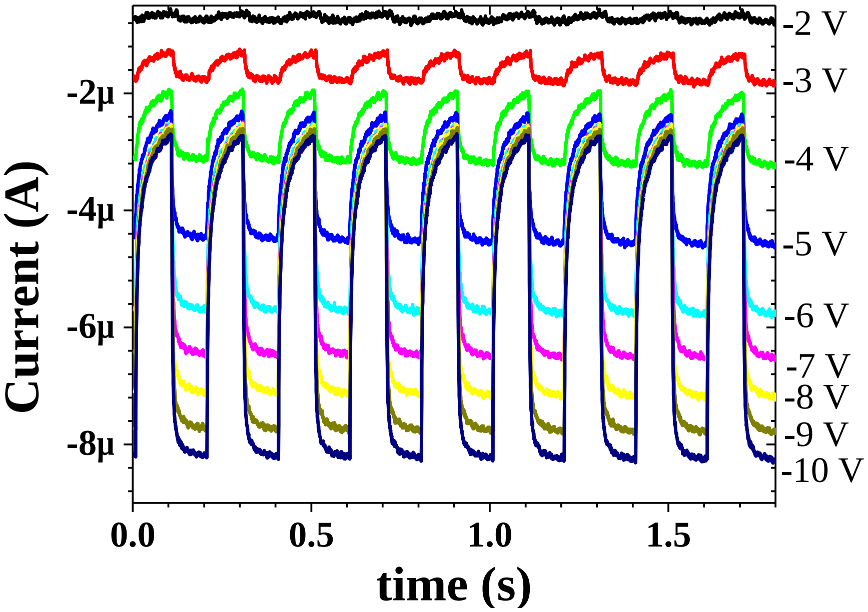

**Supplementary Figure 13.** Effect of bias voltage on the photoresponse of device D5.

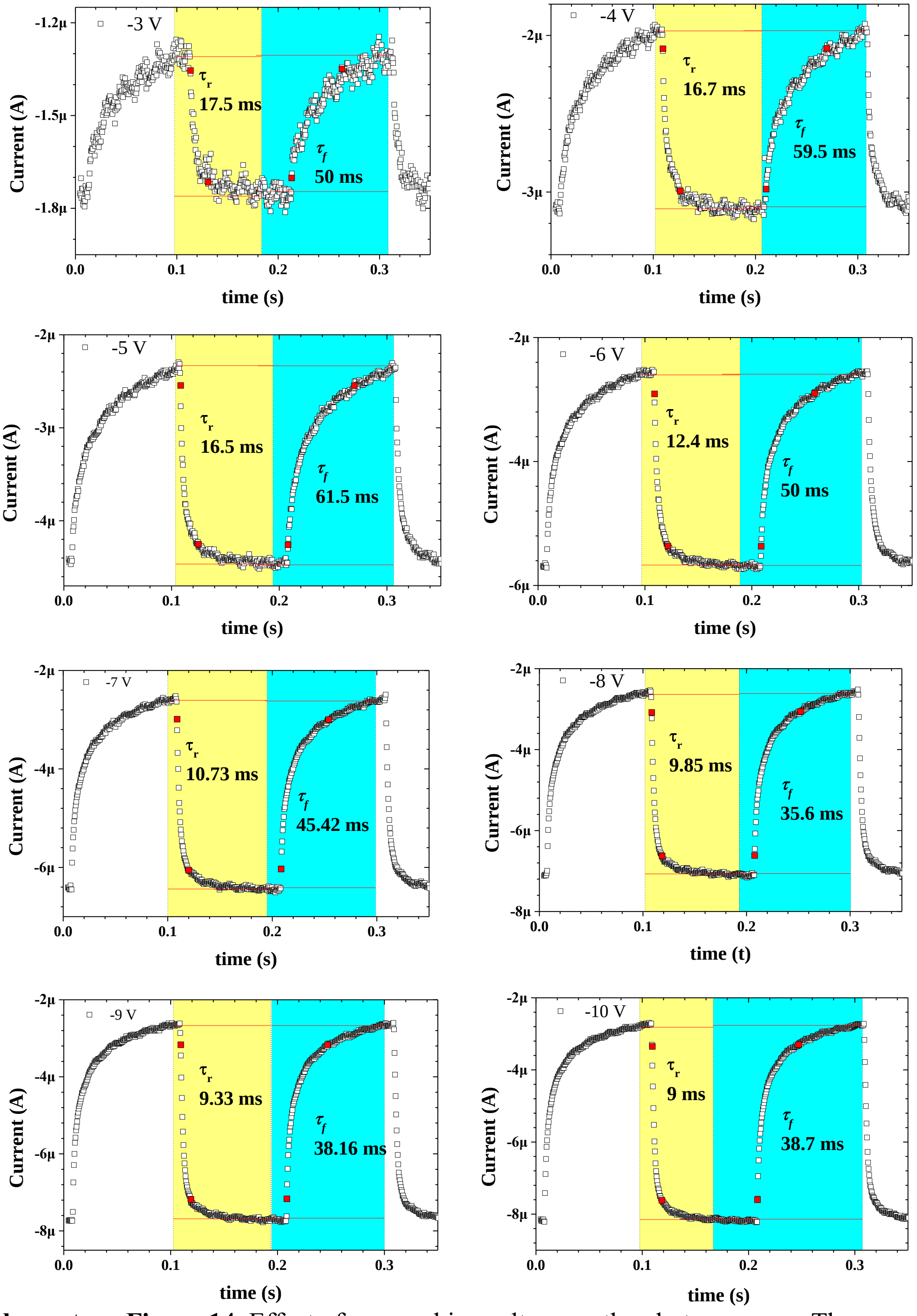


**Supplementary Figure 14.** Effect of reverse bias voltage on the photoresponse. The magnified rise and fall edges of the photoresponse of device D5 at various reverse bias conditions. The values of $\tau_r$ and $\tau_f$ of each case are estimated and provided in the respective insets.

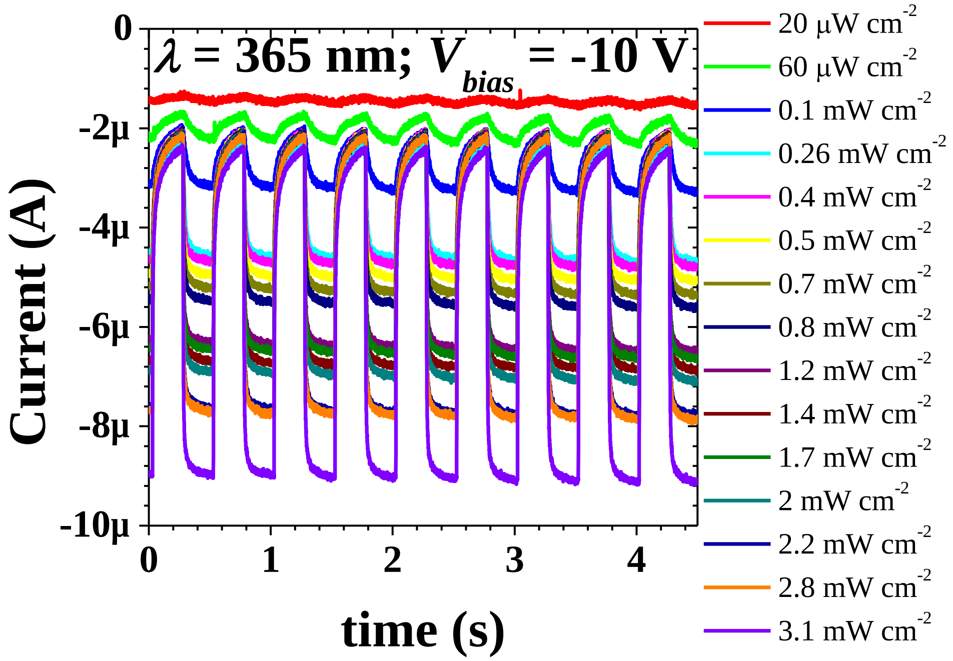


**Supplementary Figure 15.** Effect of UV light intensity on the photoresponse of device D5 at -10 V of bias voltage.

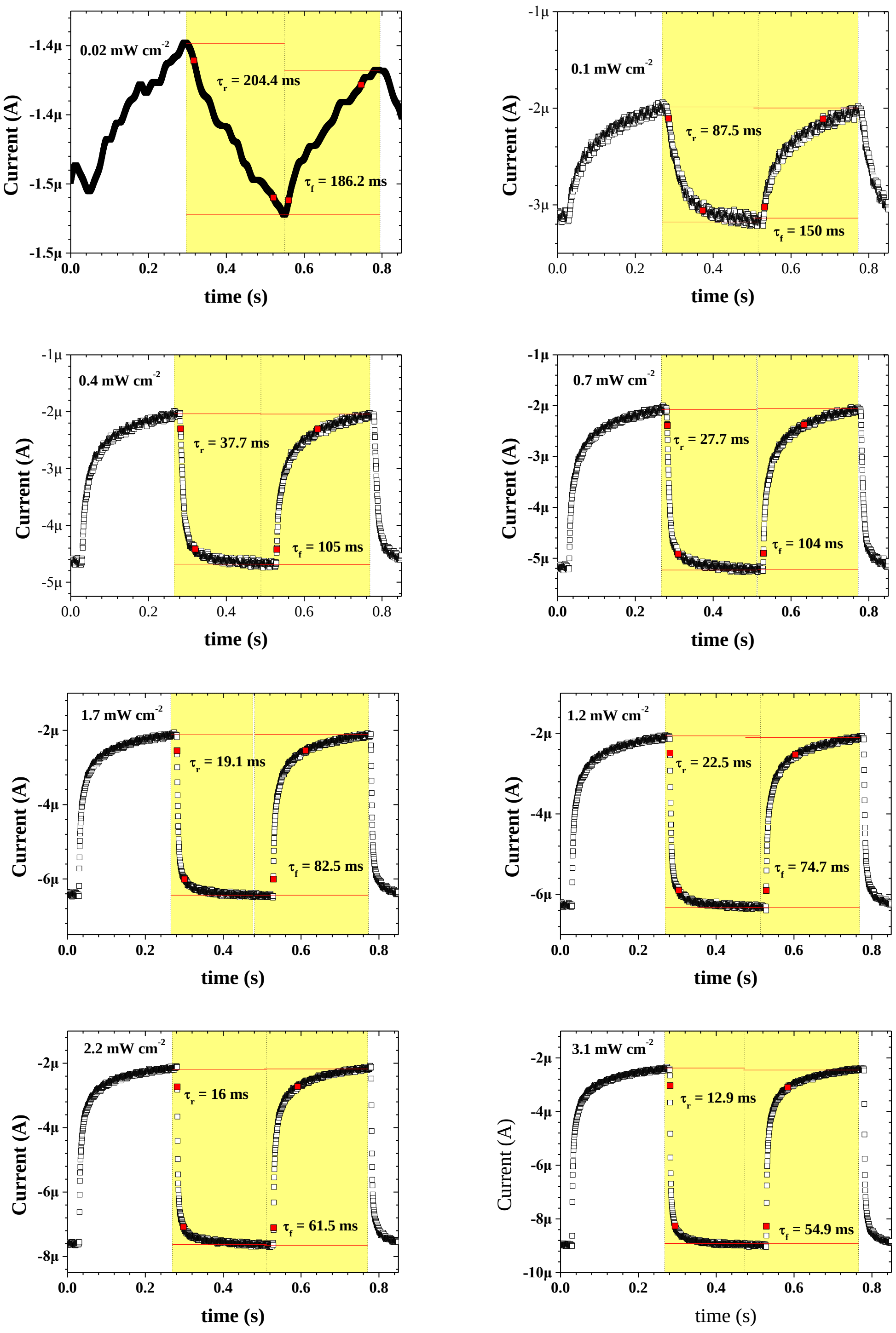


**Supplementary Figure 16.** The effect of UV light intensity on the photoresponse. The magnified rise and fall edges of the photoresponse of device D5 at various levels of light intensity from 0.02 to 3.1 mW $cm^{-2}$. The values of τr and τf of each case are estimated and provided in the respective insets. For all cases the bias voltage of -10 V was applied.

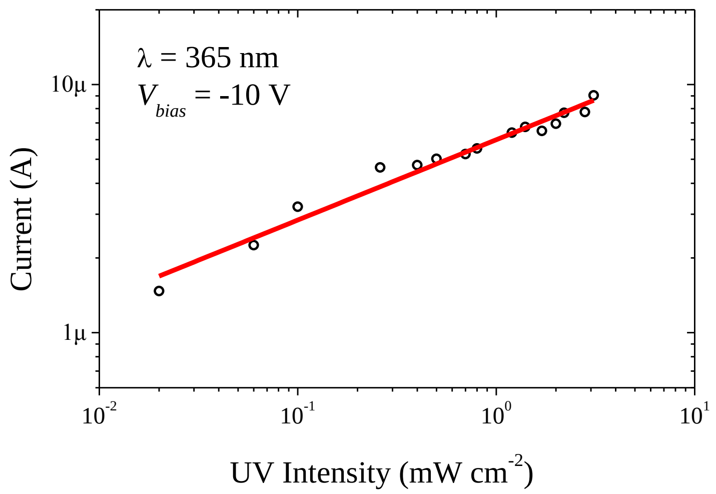


**Supplementary Figure 17.** Linear dynamic range (LDR) of the UV photodetector measured at -10 V. The total LDR is 54 dB, calculated from the relation LDR (dB) = 20 log10 ($P_{max}/P_{min}$), where $P_{max}$ and $P_{min}$ are the highest and lowest levels of incident light power for the range in which the photodetector response is linear with incident power.
